# Tough Adults, Frail Babies: An Analysis of Stress Sensitivity across Early Life-History Stages of Widely Introduced Marine Invertebrates

**DOI:** 10.1371/journal.pone.0046672

**Published:** 2012-10-12

**Authors:** M. Carmen Pineda, Christopher D. McQuaid, Xavier Turon, Susanna López-Legentil, Víctor Ordóñez, Marc Rius

**Affiliations:** 1 Department of Animal Biology (Invertebrates), University of Barcelona, Barcelona, Spain; 2 Department of Zoology and Entomology, Rhodes University, Grahamstown, South Africa; 3 Centre for Advanced Studies of Blanes (CEAB-CSIC), Blanes, Spain; 4 Department of Genetics, University of Barcelona, Barcelona, Spain; 5 Department of Evolution and Ecology, University of California Davis, Davis, California, United States of America; University of New South Wales, Australia

## Abstract

All ontogenetic stages of a life cycle are exposed to environmental conditions so that population persistence depends on the performance of both adults and offspring. Most studies analysing the influence of abiotic conditions on species performance have focussed on adults, while studies covering early life-history stages remain rare. We investigated the responses of early stages of two widely introduced ascidians, *Styela plicata* and *Microcosmus squamiger*, to different abiotic conditions. Stressors mimicked conditions in the habitats where both species can be found in their distributional ranges and responses were related to the selection potential of their populations by analysing their genetic diversity. Four developmental stages (egg fertilisation, larval development, settlement, metamorphosis) were studied after exposure to high temperature (30°C), low salinities (26 and 22‰) and high copper concentrations (25, 50 and 100 µg/L). Although most stressors effectively led to failure of complete development (fertilisation through metamorphosis), fertilisation and larval development were the most sensitive stages. All the studied stressors affected the development of both species, though responses differed with stage and stressor. *S. plicata* was overall more resistant to copper, and some stages of *M. squamiger* to low salinities. No relationship was found between parental genetic composition and responses to stressors. We conclude that successful development can be prevented at several life-history stages, and therefore, it is essential to consider multiple stages when assessing species' abilities to tolerate stress. Moreover, we found that early development of these species cannot be completed under conditions prevailing where adults live. These populations must therefore recruit from elsewhere or reproduce during temporal windows of more benign conditions. Alternatively, novel strategies or behaviours that increase overall reproductive success might be responsible for ensuring population survival.

## Introduction

Abiotic factors such as temperature, salinity and habitat characteristics have long been considered primary factors affecting survival, fitness and distribution of marine organisms [1]. More recently, anthropogenic changes to the environment have yielded new agents of selection, with resistance to pollution being one of the most important [2], [3]. Thus, the persistence of human-mediated stressors in the environment nowadays contribute to shaping the distribution of marine organisms, excluding some (e.g. [4]) and facilitating the establishment of others (e.g. [5]). Moreover, a species' long-term performance is modulated by abiotic factors across multiple life-history stages, including adulthood [6]–[9], and embryonic and larval development (e.g. [10]–[12]). Among these, embryogenesis, settlement and metamorphosis are critical life-history phases for many organisms (e.g. [13], [14]), especially when exposed to anthropogenic stressors [15]–[17]. For sessile marine organisms, where adults are unable to escape unfavourable abiotic conditions, the importance of successful early stages is even more striking as it determines the viability of local adult populations [18]–[20]. This in turn can have community-level consequences as many sessile species act as ecosystem engineers, *sensu* [21], providing habitat for multiple associated organisms while excluding competitors for space.

The arrival and establishment of non-indigenous species (NIS) via man-mediated transport is a major factor altering communities worldwide (e.g. [22], [23]). Shipping facilities such as harbours and marinas often act as entrance gates for NIS [24]–[28], and thus newcomers have to be able to cope with the stressful conditions (e.g. pollution, disturbance) that characterize these altered habitats. Establishment of NIS in such environments depends on physical and biological conditions being suitable not only for adults [29]–[31] but also for juvenile stages (e.g. [32]).

Genetic diversity is an important factor influencing the establishment of NIS [33]–[36] and it is generally assumed that the richer the genetic composition of a species' population, the wider its potential ability to adapt to stressful environmental situations [35], [37]. The heritability of traits under selection depends on stress-response variation within a population, and the potential for rapid evolution in new environments [17], [38]. For NIS, the latter can be problematic as introduced species often experience genetic bottlenecks that can reduce the genetic diversity needed for selection [35], [39], [40]. The study of genetic variability of introduced populations is essential to understanding NIS tolerance of environmental stresses and their potential to spread. To date, however, few studies have considered how different levels of parental genetic diversity in NIS influence offspring responses to multiple stressors.

Genotype-environment interactions are generally considered when differences in response between genotypes are not consistent from one environment to another, and have been investigated to assess, for instance, phenotypic stability [41] or genotypic responses to lethal and non-lethal stresses [42]. Most studies on genotype-environment interaction have analysed the influence of abiotic conditions during adulthood (e.g. [43]), while studies covering different, presumably more sensitive, early life-history stages remain rare. In line with this, genetic markers can be used to characterize different populations and to relate differences in biological response to genetic diversity and differentiation between and within populations.

Here we investigated the performance across multiple life-history stages of two widely introduced marine invertebrate species in locations where both species coexist. The solitary ascidians *Styela plicata* (Lesueur, 1823) and *Microcosmus squamiger* (Michaelsen, 1927) are sessile organisms that have been introduced worldwide [44], [45] and that often inhabit places with highly variable abiotic conditions [7], [46]–[48]. The success of introductions of *S. plicata* to new habitats has been linked to its high tolerance of polluted waters and changes in temperature and salinity [7], [10], [49], while *M. squamiger* is known to be resistant to low salinities as adults [46]. In addition, previous genetic studies of these widespread species based on a fragment of the mitochondrial gene Cytochrome Oxidase subunit I (*COI*) have revealed the existence of two highly divergent and widely distributed haplogroups for each species [44], [45]. No information is available, however, on the functional significance of this intraspecific genetic structure in terms of responses to stress. In this study, we targeted several early life-history stages (fertilization, larval development, settlement, and metamorphosis) and we genetically characterized the progenitors. We tested species performance under thermal and salinity stress, and with several concentrations of a heavy metal (Cu). We hypothesised that *S. plicata* and *M. squamiger* offspring would develop well under realistic environmental conditions found in sheltered habitats where adults occur, although different haplogroups might respond to stress differently.

## Methods

### Field sites and general methods

Adult individuals of *Styela plicata* and *Microcosmus squamiger* were collected during the austral spring of 2010 (October and November) when both species are known to reproduce [50]–[52]. Two sites along the South African coast, approximately 160 km apart, were sampled: Port Elizabeth (33°57′44″S, 25°38′8″E) and Knysna (34°02′32″S, 23°02′40″E). The selection of the sampling sites was based on the co-occurrence and abundance of the studied species and the availability of genetic data from previous studies [44], [45]. The sites feature slightly different characteristics in terms of abiotic parameters and ship traffic. Port Elizabeth harbour is an industrial port, with temperature oscillating around 16–18°C in winter and 22–22°C in summer, although there can be rapid fluctuations of up to about 10°C (range c. 14–24°C) due to periodic influx of warm water from the Agulhas Current and cooler upwelled water from nearby Cape Recife [53]. There is little freshwater input into the bay as a whole and salinities are consistently close to oceanic norms of 35.2‰ [54]. Copper concentrations in this harbour are between 0.5 µg/L and 11.3 µg/L [55]. In contrast, Knysna lagoon supports only recreational boating and can have a partially estuarine regime, with alternation of freshwater influx and tidal input. Salinities in the lagoon are generally similar to the open ocean [56], although freshwater conditions can reign in the area during unusually wet periods, displacing non-euryhaline organisms (M.R., *pers. obs.*). Temperatures generally oscillate around 15°C in winter and 22–24°C in summer. Copper levels in the waters of Knysna Lagoon range between <0.1 to 4.7 µg/L [57], [58].

After collection, individuals were transported to the laboratory (located less than 6 h away) in insulated containers containing water from the collection site. Since seawater from harbours usually contains high concentrations of pollutants [55], [57], [59], [60], we collected seawater from nearby clean sites far from any urban or industrial influence (33°58′47″S, 25°39′29″E for Port Elizabeth, 34°03′42″S, 23°22′38″E for Knysna). Animal storage and all laboratory experiments were conducted using this seawater, which we previously filtered using a vacuum filtration unit with 10 µm pore filters. Individuals were kept in the laboratory at constant temperature (20°C) and water aeration, for a minimum of 12 h and a maximum of four days for acclimatization. During the storage period in aquaria, temperature and salinity were monitored and modified to maintain the desired conditions. We used constant artificial illumination to prevent light-induced spawning [61]. All the samples were obtained according to current South African regulations. This species are not protected by any law and all sampling was conducted outside protected areas.

### Experimental trials

We chose an array of abiotic factors (temperature, salinity and pollution) that are known to influence survival of marine invertebrates [10], [46], and analysed four early life-history processes: fertilisation, development of the larvae, settlement and metamorphosis. Temperatures were set to either 20°C (control) or 30°C (treatment) in a Constant Environment (CE) room. Seawater temperature of 30°C represents the higher values occasionally reached in summer within the distributional area of both species [62]. Distilled water was added to seawater to achieve reduced salinity values (26‰ and 22‰) similar to those that are known to affect ascidian development and survival and can be found in estuaries [48], [63]. For the pollution treatments, we used copper because it is known to be one of the most toxic heavy metals for marine invertebrates [64], especially during early life-history stages [65]–[67]. We added liquid copper (Spectrosol® ref.14139 1000 ppm copper standard solution) to filtered seawater to attain the desired concentrations: 25 µg/L, mean concentration in a polluted harbour [2]; 50 µg/L, common in highly polluted harbours or near boats recently painted with antifouling paint [68] and 100 µg/L, an extreme copper concentration often used in this type of studies [69].

### Gamete extraction, fertilisation and experimentation

Gametes were extracted by dissecting the ripe gonads as described in Svane and Young [70] and Marshall et al. [71]. A mix of eggs and sperm was poured through a 100-µm filter with seawater into a small beaker to retain the eggs in the filter and gather the sperm and seawater in the beaker. For each fertilisation attempt (see Table 1 for details), around 10 individuals were dissected: 5 individuals for eggs and 5 for sperm (both species are simultaneous hermaphrodites). The oocytes obtained from the 5 female donors (around 12 to 18 ml per individual, ∼500 eggs ml^−1^) were subsequently pooled together, and the same was done with the sperm obtained from the 5 male donors (∼10^7^ sperm ml^−1^).

**Table 1:** Artificial fertilisation runs for each species and sampled site.

Species	Population	Fertil. Date	N.Indiv.	Parameters studied
*S. plicata*	Port Elizabeth	8^th^ October	10	Settlement & Metamorphosis
		16^th^ October	10	Fertilization & Larval Development
	Knysna	24^th^ October	10	All parameters
*M. squamiger*	Port Elizabeth	8^th^ October	9	Settlement & Metamorphosis
		5^th^ November	6	Fertilization & Larval Development
	Knysna	24^th^ October	10	All parameters

For the fertilization and larval development assays, 6 ml of the oocyte suspension, 12 ml of the corresponding treatment solution (filtered seawater for the temperature treatment, other treatments adjusted to obtain the desired final concentrations after mixing with gametes), and 2 ml of concentrated sperm mix were added to a 65 mm Petri dish. The cultures were then immediately taken to the appropriate CE room for fertilisation. After 1 hour, the eggs were washed with the treatment solution to remove excess sperm using a 100-µm filter and then distributed among five Petri dishes (∼100–500 eggs per dish) containing 12 ml of the treatment solution at the appropriate concentrations, and closed with a lid to avoid evaporation during the experimental period. This first set of cultures was used to assess fertilisation and development rates.

To obtain enough larvae to conduct the settlement and metamorphosis assays, new individuals were obtained from each species (Table 1) and fertilized in an aerated beaker containing 500 ml filtered seawater and maintained in a CE room at 20°C to maximize development rates [72]. Post-hatching experiments consisted of 40 larvae carefully pipetted out and placed in a Petri dish with 12 ml of the corresponding treatment solution (5 replicates per treatment and location). Petri dishes were previously submerged in seawater for 24 h to develop a biofilm in order to facilitate larval settlement [73], [74]. All Petri dishes were then placed in CE rooms (30°C for the temperature treatment and 20°C for the rest of experimental conditions) and kept for 4 days.

### Data collection and analyses

**Figure 1:**
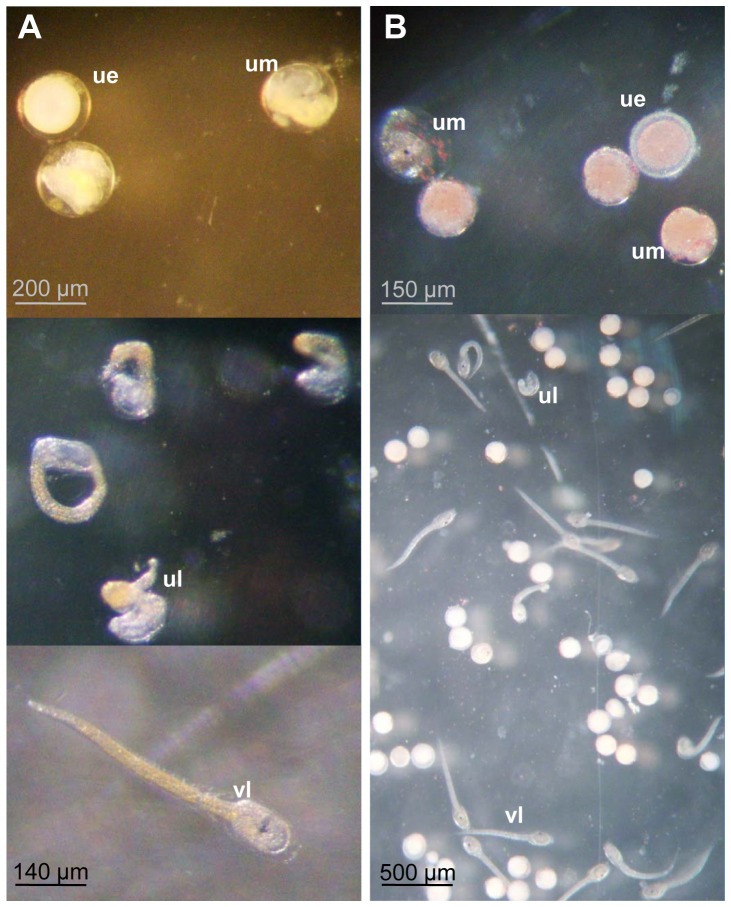
Picture of eggs and larvae under stereomicroscope. a) *S. plicata* and b) *M. squamiger* (ue: unfertilized egg; um: undeveloped embryo; ul: unviable larvae; vl: viable larvae).

For both species, most of the larvae hatched within 14 hours of fertilisation at 20°C. Numbers of viable larvae, larvae with deformities (or immature larvae), undeveloped embryos and unfertilized eggs (Fig. 1) were then recorded using a stereomicroscope. Likewise, the numbers of settled, completely metamorphosed and unattached larvae were assessed every 24 h over 4 days (96 h) in the settlement and metamorphosis assays. The fertilisation rate (FR), development rate (DR), settlement rate (SR) and metamorphosis rate (MR) were calculated as follows:
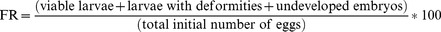


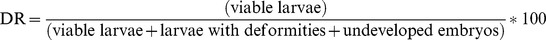


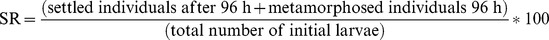


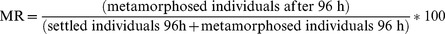
We analyzed two types of variables, the proportion of success at each developmental stage (i.e. fertilization rate, development rate, settlement rate, and metamorphosis rate) for controls and treatments, and the relative success ratios (RS) obtained by dividing the value of each rate by the mean of the corresponding controls. The former was used to assess treatment effects against the controls. For site effects, as differences between sampled sites often occurred even in the controls, the RS were an appropriate assessment of the effect of interest (i.e., whether development was impaired differentially in one site with respect to the other, after eliminating the effect of differences in controls).

For both types of variables, we performed separately two-way analyses of variance (ANOVA) per species with site and treatment as fixed factors. We used a logit transformation of the FR, DR, SR and MR data as it is known to stabilize the variances of proportional data better than other commonly used methods [75]. Our transformed data had homogeneity of variances in all datasets, although normality was only accomplished in a few cases. Nonetheless, we performed the ANOVA tests as they are robust to departures from normality when variances are homogeneous [76]. For the relative success rates (RS), the data complied in all cases with the homoscedasticity assumption, although they weren't normally distributed in some cases. As several transformations tried did not improve this, we proceeded with the raw data in the analyses.

For the proportion data, used to assess treatment effects, if the interaction between factors was significant, post-hoc analyses of treatments were performed at each site against the control with Dunnett's test. If the interaction was not significant, post-hoc tests on treatment levels were done combining both sites. For the RS variables, used to determine site differences, when interaction was significant, site effects were assessed within each level of treatment (using a post-hoc Student-Newman-Keuls test). If interaction was not significant, no test was necessary as site had only two levels. In all post-hoc analyses, the residual mean square obtained from the original two-way ANOVAs was used to calculate the standard errors of the means for the post-hoc comparisons [76], [77]. Statistical analyses were performed using the software STATISTICA v. 6.1 (©StatSoft, Inc. 1984–2004).

In order to obtain an overall estimate of success (from egg fertilisation to post-metamorphic formation), we also calculated the cumulative % success of the different stages, for each of the treatments. For this purpose, each of the different rates (FR, DR, SR, MR) was multiplied by the mean of the previous stage.

### Screening of parental genotypes

A piece of muscular tissue from the mantle or the siphon of each individual used for fertilisation was dissected and immediately preserved in absolute ethanol (Table 2). After a few hours, the tainted ethanol was replaced by new absolute ethanol and samples were then stored at −20°C until extracted. Total DNA was extracted using the REDExtract-N-Amp Tissue PCR Kit (Sigma-Aldrich). The universal primers LCO1490 and HCO2198 described in Folmer et al. [78] were used to amplify a fragment of the *COI* gene (maternally inherited). Amplifications were performed in a final volume of 20 µL using 10 µL of REDExtract-N-amp PCR reaction mix (Sigma-Aldrich), 0.8 µL of each primer (10 µM), and 2 µL of template DNA. The PCR program consisted of an initial denaturing step at 94°C for 2 min, 30 amplification cycles (denaturing at 94°C for 45 seconds, annealing at 50°C for 45 seconds and extension at 72°C for 50 seconds), and a final extension at 72°C for 6 min, on a PCR System 9700 (Applied Biosystems). PCR products were sent for purification and sequencing to Macrogen Inc. (Seoul, Korea). Sequences were edited and aligned using BioEdit® v.7.0.5.3 [79]. Number of alleles (*Nh*), gene diversity (*Hd*), and nucleotide diversity (*π*) were computed with DnaSP v.5 [80]. Pairwise genetic distances (*F*_ST_) using allele frequencies were calculated with Arlequin v.3.1 [81] and their significance was assessed by performing 10,000 permutations. Note that because each fertilization attempt involved a combination of gametes from ten different donors, offspring resulted from a random combination of these genotypes.

**Table 2:** Diversity measures and population differentiation values (*F*_ST_) for the mtDNA sequences (*COI* gene).

*Species*	*Pop.*	*N*	*Nh*	*Hd*	±SD	*π*	±SD	*Haplotypes*	*Lineage*	*Fst*	*p-value*
*S. plicata*	PE	20	2	0.100	(±0.088)	0.00292	(±0.00257)	Hap 2 (0.5)	*I*	0.7278	<0.001
								Hap 5 (0.95)	*II*		
*S. plicata*	KN	10	2	0.556	(±0.075)	0.00095	(±0.00013)	Hap 1 (0.5)	*I*		
								Hap 2 (0.5)	*I*		
*M. squamiger*	PE	13	6	0.769	(±0.103)	0.0035	(±0.00173)	Hap 7 (0.08)	*I*	−0.048	0.991
								Hap 53 (0.08)	*I*		
								Hap 1 (0.46)	*II*		
								Hap 5 (0.23)	*II*		
								Hap 9 (0.08)	*II*		
								Hap 23 (0.08)	*II*		
*M. squamiger*	KN	10	6	0.844	(±0.103)	0.00495	(±0.00257)	Hap 14 (0.1)	*I*		
								Hap 1 (0.40)	*II*		
								Hap 5 (0.20)	*II*		
								Hap 54 (0.1)	*II*		
								Hap 55 (0.1)	*II*		
								Hap 56 (0.1)	*II*		

Mitochondrial lineages according to Rius et al. [44] and Pineda et al. [45]. Number of individuals analyzed per population (*N*).

Number of haplotypes per population (*Nh*), Haplotypic (*Hd*) and nucleotidic (*π*) diversity, and their corresponding standard deviations in brackets. Pairwise genetic distances (*F*_ST_).

## Results

### Experimental trials

**Figure 2:**
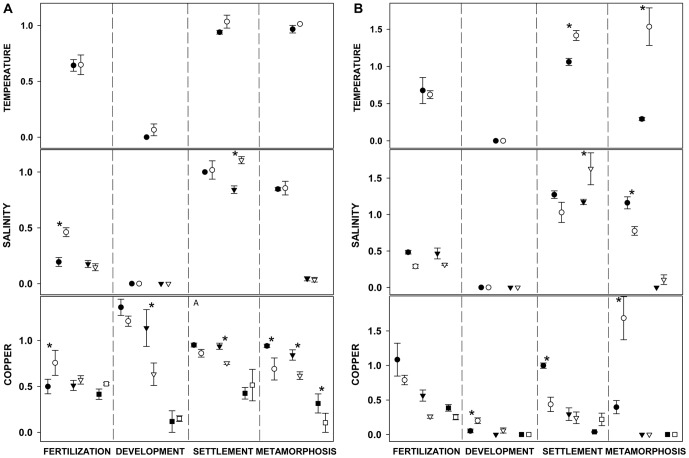
Relative success ratios of each developmental stage. a) *S. plicata* and b) *M. squamiger*. Values are relative to the controls (i.e., values<1 indicate less success than in the corresponding controls). Treatments include: temperature (30°C), salinity (circles for 26‰ salinity, triangles for 22‰) and copper (circles for copper concentration of 25 µg/L, triangles for 50 µg/L and squares for 100 µg/L). Black symbols correspond to the population at Port Elizabeth, while white symbols correspond to the population at Knysna. Values are means ±standard errors. Asterisks indicate significant differences between locations.

The results of the ANOVA on the different logit-transformed rates are presented in Tables 3 and 4. The results of post-hoc site comparisons using relative success rates are presented in Fig. 2, where these rates are depicted. All abiotic conditions analyzed: temperature at 30°C, salinity values of 22‰ (22S) and 26‰ (26S), and copper at a concentration of 25 µg/L (Cu25), 50 µg/L (Cu50) and 100 µg/L (Cu100), produced important effects on the relative success ratio of each developmental stage considered, with differences due to both species and the site of adult collection. There was no consistent trend of one of the sites having higher or lower success rates, although many outcomes differed significantly between sites (Tables 3,4; Fig. 2).

**Table 3:** ANOVA examining the effects of site and treatment at four developmental stages for *S. plicata*.

Source	*df*	MS	*F*	*P*
**Effect on the FERTILISATION Rate**				
Site	1	0.149	1.276	0.263
Treatment	6	4.063	34.903	<0.001
Site x Treatment	6	0.37	3.179	0.009
Error	56	0.116		
Comparisons for factor Treatment within site (Dunnett test, p = 0.05)				
Port Elizabeth	T, 26S,22S,Cu25,Cu50,Cu100<Control			
Knysna	26S,22S,Cu50, Cu100<Control			
**Effect on the DEVELOPMENT Rate**				
Site	1	31.451	20.438	<0.001
Treatment	6	135.769	88.230	<0.001
Site x Treatment	6	5.332	3.465	0.006
Error	56	1.539		
Comparisons for factor Treatment within site (Dunnett test, p = 0.05)				
Port Elizabeth	T,26S,22S,Cu100<Control			
Knysna	T,26S,22S,Cu100<Control<Cu25			
**Effect on the SETTLEMENT Rate**				
Site	1	115.035	74.075	<0.001
Treatment	6	17.786	11.453	<0.001
Site x Treatment	6	9.873	6.358	<0.001
Error	42	1.553		
Comparisons for factor Treatment within site (Dunnett test, p = 0.05)				
Port Elizabeth	T,22S,Cu25,Cu50,Cu100<Control			
Knysna	No differences			
**Effect on the METAMORPHOSIS Rate**				
Site	1	3.256	1.246	0.271
Treatment	6	86.937	33.256	<0.001
Site x Treatment	6	12.731	4.870	<0.001
Error	42	2.614		
Comparisons for factor Treatment within site (Dunnett test, p = 0.05)				
Port Elizabeth	22S,Cu100<Control			
Knysna	.26S, 22S,Cu25,Cu50,Cu100<Control			

T: temperature at 30°C; 22S: 22‰ salinity; 26S: 26‰ salinity, Cu25: copper concentration of 25 µg/L;

Cu50: 50 µg/L; and Cu100: 100 µg/L.

**Table 4:** ANOVA examining the effects of site and treatment at four developmental stages for *M. squamiger*.

Source	*df*	MS	*F*	*P*
**Effect on the FERTILISATION Rate**				
Site	1	0.062	0.881	0.353
Treatment	6	1.912	27.295	<0.001
Site x Treatment	6	0.156	2.231	0.059
Error	42	0.070		
Comparisons for factor Treatment (Dunnett test, p = 0.05)				
	T,26S,22S,Cu50,Cu100<Control			
**Effect on the DEVELOPMENT Rate**				
Site	1	7.379	7.300	0.010
Treatment	6	69.155	68.415	<0.001
Site x Treatment	6	2.108	2.086	0.075
Error	42	1.011		
Comparisons for factor Treatment (Dunnett test, p = 0.05)				
	T,26S,22S,Cu25,Cu50,Cu100<Control			
**Effect on the SETTLEMENT Rate**				
Site	1	10.900	21.621	<0.001
Treatment	6	28.538	56.610	<0.001
Site x Treatment	6	3.789	7.517	<0.001
Error	56	0.504		
Comparisons for factor Treatment within site (Dunnett test, p = 0.05)				
Port Elizabeth	Cu50,Cu100<Control<26S			
Knysna	Cu25,Cu50,Cu100<Control<22S			
**Effect on the METAMORPHOSIS Rate**				
Site	1	0.782	1.362	0.248
Treatment	6	100.818	175.607	<0.001
Site x Treatment	6	5.648	9.839	<0.001
Error	56	0.574		
Comparisons for factor Treatment within site (Dunnett test, p = 0.05)				
Port Elizabeth	T,22S,Cu25,Cu50,Cu100<Control			
Knysna	22S,Cu50,Cu100<Control			

T: temperature at 30°C; 22S: 22‰ salinity; 26S: 26‰ salinity, Cu25: copper concentration of 25 µg/L;

Cu50: 50 µg/L; and Cu100: 100 µg/L.

#### *S. plicata*

There were significant interactions of treatment and site for all dependent variables. *S. plicata* showed significantly reduced fertilisation rates (FR) in most treatments (Table 3). Knysna gametes seemed somewhat less affected by the treatments than Port Elizabeth (Fig. 2a). All treatments had significant effects in Port Elizabeth, while gametes from Knysna were unaffected by temperature and Cu25 (Table 3). Significant site differences were found for 26S and Cu25, where fertilization relative to controls was significantly higher in Knysna (Fig. 2a).

The development of viable larvae (DR) was probably the most sensitive stage in the early development of this species (Fig. 2a) and was significantly impaired by all treatments, except for Cu25 and Cu50 (Table 3). Notably, the presence of Cu25 increased DR relative to the controls (thus relative success rates were above 1), although the effect is significant only in Knysna. Significant inter-site differences in relative success rates were found only for Cu50, with embryos from Port Elizabeth being more resistant.

**Figure 3:**
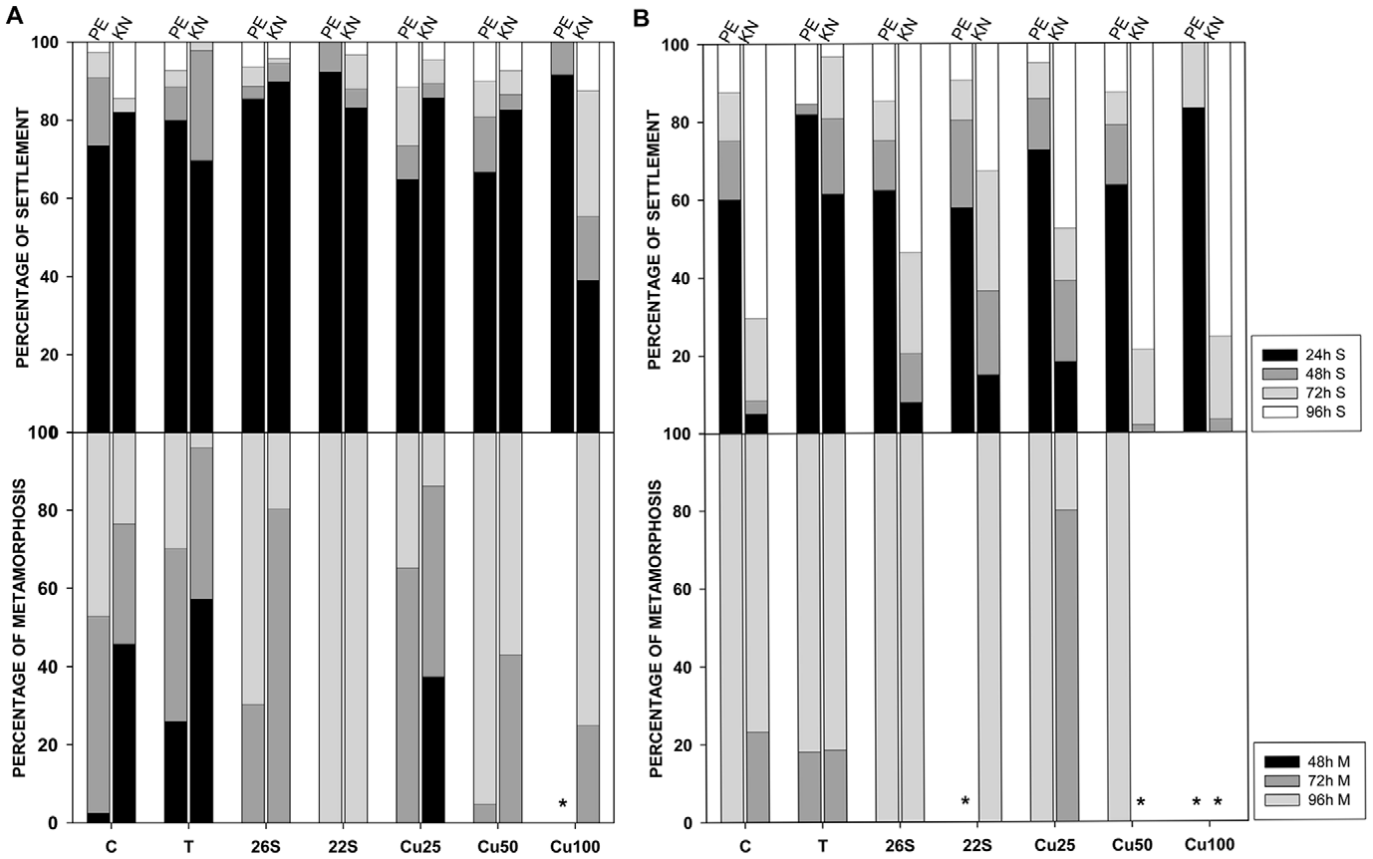
Percentage of total settled (above) and metamorphosed (below) individuals each 24-hour interval. a) *S. plicata* and b) *M. squamiger*. Left bars for Port Elizabeth (PE); right bars for Knysna (KN). Asterisks indicate zero success.

Settlement rate (SR) tended to show higher relative success values (Fig. 2a) than the previous variables, indicating that this stage is somewhat more tolerant. All treatments except 26S yielded significantly low values for Port Elizabeth larval settlement, while no significant effect was detected for Knysna (Table 3). Relative success values in Port Elizabeth were significantly lower for 22S, and significantly higher for Cu50. Although the effect of salinity (26S) on SR was not significant, low salinities did appear to accelerate settlement within 24 hours (Fig. 3a). On the other hand, Cu100 seemed to accelerate settlement for larvae from Port Elizabeth adults but to delay it for Knysna (Fig. 3a).

As for settlement, metamorphosis in *S. plicata* (MR) was also a relatively tolerant process under most treatments and for both sites. The strongest inhibition effect on MR occurred at 22S and Cu100 for both sites (Fig. 2a), and these treatments yielded significantly lower metamorphosis than the controls at both sites (Table 3). In addition, the metamorphosis of Knysna larvae was also impaired at 26S, Cu25 and Cu50 (Table 3). Site differences were significant in the three copper treatments, with relative success rates higher in Port Elizabeth. Increased temperature accelerated the metamorphosis of the settled individuals within 72 hours, although low salinities had the opposite effect, causing a delay in metamorphosis (Fig. 3a). Most of the larvae from the 22S and Cu100 treatments never achieved complete metamorphosis within 96 hours, and none did so in Port Elizabeth at Cu100 concentration (Fig. 3a).

#### *M. squamiger*

All treatments except Cu25 significantly reduced the fertilisation rates (Fig. 2b, Table 4) at both sites combined (no significant interaction term), and the most drastic reduction was observed after exposure to 26S, 22S, Cu50 and Cu100 (Fig. 2b). For the relative success rates (RS), the interaction term was not significant, and there was an overall effect of site, with mean success rates higher in Port Elizabeth.

As for *S. plicata*, larval development was the most sensitive stage (Fig. 2b). The interaction was not significant and, combining sites, all treatments significantly reduced DR, especially high temperature, 26S, 22S, Cu50 and Cu100 (Table 4, Fig. 2b). When analysing relative success rates, the interaction proved significant, and this was due to the outcome of the Cu25 treatment, being significantly higher in Knysna.

Settlement was also less affected by temperature and salinity treatments than the previous processes (Fig. 2b). The three copper concentrations resulted in significantly lower SR than the controls in Knysna, while only Cu50 and Cu100 reduced settlement of larvae from Port Elizabeth (Table 4). High temperatures and low salinities increased the number of settlers relative to the controls (values above 1, Fig. 2b), with a significant positive effect for Knysna larvae kept at 22S and Port Elizabeth larvae at 26S (Table 4). Significant site differences in relative success rates were found for temperature and 22S (higher rates in Knysna), and Cu25 (higher rates in Port Elizabeth, Fig. 2b). Moreover, Settlement was accelerated at higher temperature (Fig. 3b), while Cu50 and Cu100 delayed settlement of larvae from Knysna but not from Port Elizabeth (Fig. 3b).

All treatments except 26S significantly decreased the MR from Port Elizabeth larvae, while only 22S, Cu50 and Cu100 impaired metamorphosis of Knysna larvae (Table 4). On the other hand, more larvae metamorphosed at high temperature and Cu25 than in the controls in Knysna (leading to relative rates higher than one, Fig. 2b), although this outcome was not significant. The relative success rates were significantly higher in Knysna for temperature and for Cu25, and in Port Elizabeth for 26S (Fig. 2b). Cu25 also accelerated the timing of metamorphosis at Knysna (Fig. 3b). No metamorphosis was observed for larvae from Port Elizabeth subjected to the 22S treatment, larvae from Knysna at Cu50, or larvae from either sampled site at Cu100 (Fig. 3b).

#### *S. plicata* and *M. squamiger* comparison

**Figure 4:**
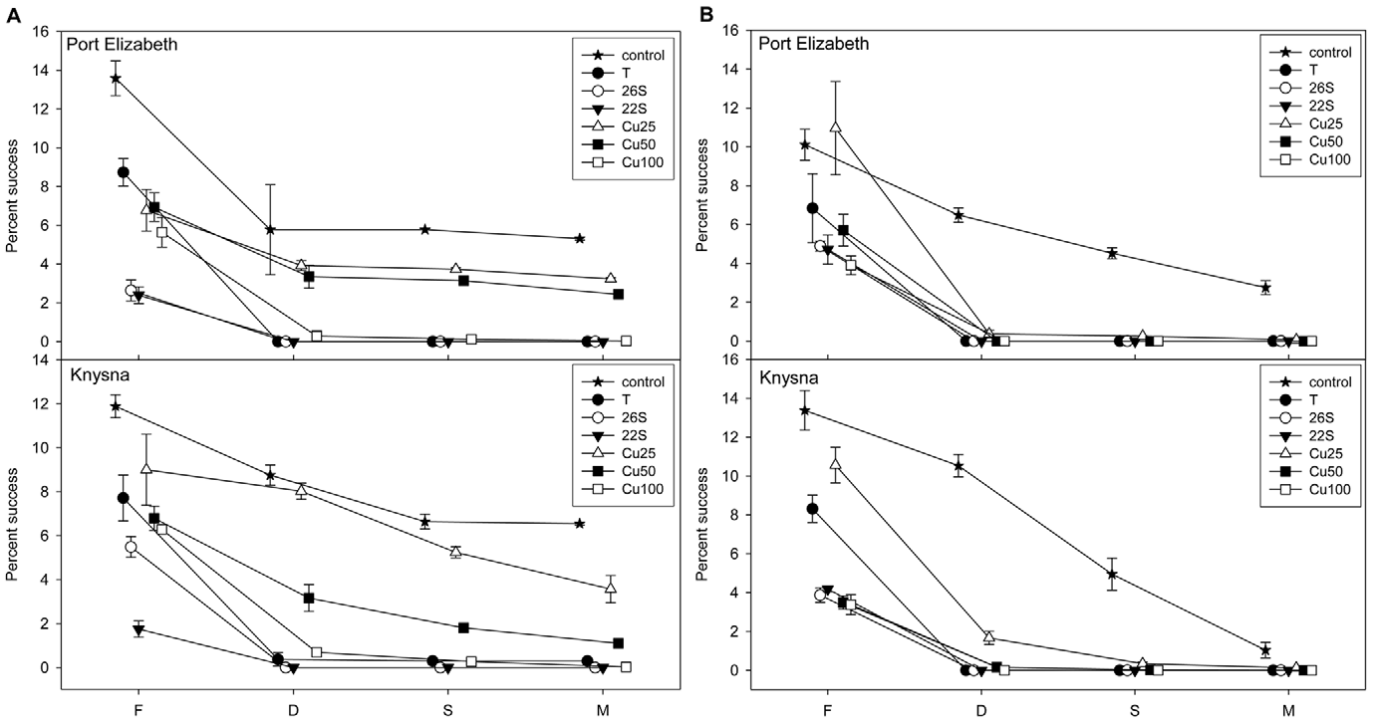
Cumulative success over fertilisation (F), development (D), settlement (S), and metamorphosis (M) for each treatment. a) *S. plicata* and b) *M. squamiger*. Legend: T: temperature at 30°C; 22S: 22‰ salinity; 26S: 26‰ salinity, Cu25: copper concentration of 25 µg/L; Cu50: 50 µg/L; and Cu100: 100 µg/L.

When the whole developmental sequence was considered, from fertilisation of the egg to post-metamorphic juveniles, clear differences in cumulative success were found between the species, with *S. plicata* being overall more tolerant of harsh conditions than *M. squamiger* (Fig. 4). As previously stated, the development of larvae seems to be the most sensitive stage for both species, acting as a bottleneck that results in a sharp reduction in the number of viable larvae in most treatments (Fig. 4).

It is particularly relevant that the complete process of reproduction and recruitment only occurred in non-negligible numbers in the controls and the treatments with the lower copper concentrations assayed (Cu25, Cu50) in *S. plicata* (Fig. 4a), and only for the controls in the case of *M. squamiger* (Fig. 4b). In all other treatments, failure of one step or another (particularly development of larvae) prevented successful completion of the early life-history stages completely or almost so.

### Genetic screening

All adults used for the fertilization experiments were sequenced (Tables 1, 2), except for two individuals of *M. squamiger* that failed to amplify. Three haplotypes were obtained for *S. plicata*, corresponding to haplotypes already described by Pineda et al. [45]. For *M. squamiger*, we found ten haplotypes. Six of these had previously been reported [44], while the sequences of the remaining four haplotypes (Hap 53–56) were new and were deposited in GenBank with accession numbers JQ815436-JQ815439 (Table 2). *S. plicata* showed two clear groups of haplotypes, with Knysna composed entirely of Lineage I (50% Hap 1 and 50% Hap 2) and Port Elizabeth mainly represented by Lineage II (95% Hap 5, 5% Hap 2), *sensu* [45]. Thus, although these three haplotypes are globally distributed [45], Port Elizabeth and Knysna were highly differentiated (*F*_ST_ = 0.728, *P*<0.001) (Table 2). Regarding *M. squamiger*, the two most frequent haplotypes were Haps 1 and 5 (Table 2) for both populations, and together represented ca. 60% of the genetic pool. Haplotypes corresponding to Lineage II, *sensu* [44], represented around 90% of each population, and the two populations did not differ significantly (*F*_ST_ = 0.048, *P* = 0.991) (Table 2).

## Discussion

Increased temperature, decreased salinity and elevated copper concentrations affected several life-history stages of the introduced ascidians *Styela plicata* and *Microcosmus squamiger* at the two studied sites. Differences according to sensitivity to abiotic stressors and life-history stages were observed but overall, fertilisation and larval development were the most sensitive stages for both species. Thus, although later stages (settlement and metamorphosis) seemed in general more tolerant, the initial stages (fertilisation and development) must necessarily happen under more benign conditions.

A few of the stressors had apparent positive effects on some stages (resulting in the corresponding rates being greater than in the controls, or accelerating processes). It has been reported that moderate concentrations of pollutants can enhance some early life-history stages of marine invertebrates but eventually lead to detrimental effects (e.g. [82], [83]). Similarly, our combined rates show that, notwithstanding these rare positive effects, the overall effect through the developmental stages considered is negative in all cases. Therefore, considering a single stage independently can lead to misleading conclusions about the ability of a species to overcome stressful conditions during the early life-history stages.

In general, *S. plicata* was more resistant to copper pollution, and both species coped similarly with increased temperature. Decreased salinity prevented complete development in both cases; however, some stages of *M. squamiger* (e.g. fertilization, settlement) are less affected or enhanced by low salinities. These tolerances correlate well with the types of environments where these species are commonly found. *S. plicata* is often found in harbours, which are known to accumulate copper [15], [45], and *M. squamiger* in estuaries, which are characterized by frequent salinity changes [84]. In fact, Lowe [46] found that adults of *M. squamiger* could withstand reduced salinity levels (15–25‰) for extended periods of time, outcompeting native species such as *Molgula manhattensis* in southern California harbours. Similarly, estuarine sites along the southeast coast of South Africa (e.g. Port Alfred, Bushman's River Mouth and East London) are dominated by *M. squamiger* while *S. plicata* is consistently absent in estuarine conditions but found in nearby harbours (M.R., *pers. obs.*).

Sensitivity differences according to development stages and stressors have been observed across phyla for other marine invertebrates, including molluscs [1], [14], echinoderms [85] and ascidians [86]–[88]. Our results indicate that complete development, from fertilisation to metamorphosis, is impaired by all studied stressors, affecting several early life-history stages. In fact, we recorded completion of early stages only in *S. plicata* if copper concentrations were at/below 50 µg/L. Thus, the wide distribution of these species in environments where high temperature, low salinity or extreme pollutant concentrations are present cannot be inferred from laboratory or manipulative studies, but must be explained by novel strategies or behaviours in nature that increase overall reproductive success [89]. In this sense, Bellas et al. [65] suggested that the ascidian *Ciona intestinalis* could probably detect trace metals in the water with the adhesive papillae and delay or inhibit attachment. Although increasing the swimming period may decrease the probability of post-settlement survival due to the high metabolic cost required for the latter [10], [90]–[92], the successful settlement and survival of a few individuals could result in successful introductions to new habitats. Even if recruitment failures were a common outcome, the prolonged reproductive period observed for both species [50]–[52] would increase the chances of a propagule finding favourable temporal windows of tolerable conditions.

The sensitivity of *S. plicata* embryos and larvae to temperature and salinity changes was in accordance with Thiyagarajan and Qian [10], who studied *S. plicata* in Hong Kong and reported recruitment failure when seawater temperature reached values of 26–30°C and salinities of 22–30‰ in summer. In our study, these conditions prevented both *S. plicata* and *M. squamiger* from completing development, with the earlier stages (embryo fertilisation and larval development) being especially sensitive, while settlement was hardly affected. The lowest salinity tested (22‰), however, prevented most larvae of either species to complete metamorphosis even after successful settlement, as previously described for other ascidians [63], [93]. High temperatures and low salinities also tended to accelerate development, with most larvae of both species settling within 24 h, which would limit options for escape to more favourable sites. Thus, the current climate change predictions of increasing temperatures and decreasing salinities [94] suggest that these species, particularly *S. plicata* [48], would find it difficult to cope with these predicted stresses [95].

Copper has been shown to inhibit embryo development, reduce successful settlement and metamorphosis, and reduce growth in many marine invertebrates, including ascidians (e.g. [17], [96]–[98]). Elevated copper concentrations also negatively affected the early life-history stages of *S. plicata* and *M. squamiger*, with more dramatic effects on developmental stages of the latter species. Even at copper concentrations similar to those found in highly polluted harbours (25–50 µg/L), fertilisation success of *S. plicata* was still around 50% that of the controls for both populations, and development through metamorphosis was possible. At the highest concentration (100 µg/L), though, there was no development of the larvae and the metamorphosis of settled individuals was seriously impaired. In contrast, even the lowest concentration of copper assayed (25 µg/L) had detrimental effects on early development of *M. squamiger*. This suggests that *S. plicata* will continue to perform better in polluted habitats than *M. squamiger* and has important implications for understanding the distributions of the two species across overlapping ranges.

The genetic patterns found were clear-cut: genetic differentiation was high between populations of *S. plicata*, while it was negligible for *M. squamiger*. However, we could not detect a clear correlation of this pattern with differential responses to abiotic stress. In general, although some particular outcomes were significantly different, all populations responded similarly to the tested stressors. Genetic diversity within populations was lower for *S. plicata* than for *M. squamiger*, but again this has no clear connection with our results as, if any, the low genetic diversity species *S. plicata* was overall more tolerant to stress than *M. squamiger*. The only emerging pattern was found when comparing the responses to low salinity and high copper concentrations between populations of *S. plicata*. For instance, fertilization rates at low salinities (26‰) were considerably higher for the eggs from Knysna than for the eggs from Port Elizabeth. Adult samples from Knysna exclusively displayed haplotypes from Lineage I, which is the most widespread haplogroup in the world [45]. In contrast, adults from Port Elizabeth mainly belonged to Lineage II, which is also found in salt marsh habitats [48]. Thus, the slightly different response of these two populations of *S. plicata* may be related to differences in their genetic composition. Differential adaptation to environmental factors (e.g. temperature, salinity) of mitochondrial sequences within one species has been previously described in marine invertebrates [99]–[103]. Of course this adaptive capability need not be directly linked to the studied gene, but can be related to other genes that vary between lineages. In order to assess whether there is any genetic basis in the responses featured by both species, a more precise genetic characterization (for example, using microsatellites), together with controlled crossings and transplant experiments are necessary.

In conclusion, we found that several early life-history stages of the ascidians *S. plicata* and *M. squamiger* were seriously impaired by exposure to realistic scenarios of abiotic stressors, independent of the haplogroup tested. Moreover, abiotic factors do not affect animals in isolation but will normally combine as multiple stressors, often resulting in additive or synergistic effects. Thus, our results are likely to overestimate the resilience of the life-history processes studied here, a surprising fact given the abundance of these species in habitats such as harbours where such stressors are the norm. Behavioural strategies that can only be observed in the field (e.g. delay in spawning until suitable conditions are restored, strong propagule pressure with arrival of larvae from more benign environments, extended reproductive periods) seem plausible explanations for the presence of adults in these localities. Basic knowledge of reproduction, larval development and survival of these species in new habitats coupled with further information on their genetic variability is therefore essential to predict possible areas of establishment and spread worldwide.
